# Pattern of Chronic Venous Insufficiency among Patients Presenting to a Vascular Surgery Clinic in Low- to Middle-Income Countries (LMIC): A Cross-Sectional Study

**DOI:** 10.3400/avd.oa.20-00088

**Published:** 2021-06-25

**Authors:** Zia Ur Rehman

**Affiliations:** 1 Section of Vascular Surgery, Department of Surgery, Aga Khan University Hospital, Karachi, Pakistan

**Keywords:** chronic venous disease, clinical, etiological, anatomic and pathophysiologic classification, varicose veins, frequency, risk factors

## Abstract

**Objective**: To measure the pattern and severity of chronic venous insufficiency (CVI) in patients presenting to a vascular surgery clinic in Pakistan.

**Materials and Methods**: This cross-sectional study has examined patients presenting with CVI for the first time. Patients were assessed for severity of the disease using clinical, etiological, anatomical and pathological (CEAP) score and venous clinical severity score (VCSS). Patients were then divided into two groups depending on the severity of the disease: ‘mild/moderate’ CVI if the CEAP classification was ≤C3 or VCSS was <5 and ‘severe’ if CEAP classification was >3 or VCSS was ≥5. Both groups were then compared to determine the factors associated with the more ‘severe’ form of CVI.

**Results**: During the study duration, 121 patients presented with CVI with mean age of 47.83±12.02 years; 74 (61.2%) were female. Mean body mass index of the patients was 32.49±18.3 kg/m^2^. Mean VCSS was 5.49±3.84, indicating most patients presented with the severe form of CVI. Field workers were determined to be three to five times more likely to present with severe CVI compared to housewives and office workers.

**Conclusion**: Majority of the patients who presented to a tertiary care facility had the severe form of CVI. Thus, there is a need to raise awareness on this disease at community level.

## Introduction

Chronic venous insufficiency (CVI) is one of the most common diseases worldwide. Its prevalence ranges from 10% to 30%.^1,2)^ It is a progressive disease caused by venous hypertension secondary to venous reflux or obstruction. Clinical presentations of CVI range from mild skin trophic changes to non-healing venous ulcers.^3)^ The prevalence of CVI is highest in Western countries where it already consumes up to 2% of healthcare.^4)^ The symptoms are often ignored by the patient until it gets debilitating, hindering one’s day-to-day social and occupational activities. Patients usually present late with advance form of disease. There is limited information available regarding the severity of CVI in our population. Thus, this study aimed to determine the pattern of CVI in patients presenting to a tertiary care hospital and to identify risk factors associated with the more severe form of the disease.

## Materials and Methods

This cross-sectional study was conducted at vascular surgery clinics in Aga Khan University Hospital, Karachi, for 6 months (from 1st October 2019 to 31st March 2020). Patients were selected by non-probability consecutive sampling technique. Patients presenting for the first time with CVI either due to primary, secondary or recurrent varicose veins were included in this study. Patients with leg pain, swelling and ulcer of non-venous pathology were excluded from analysis. Patients were assessed using two scoring systems: clinical, etiological, anatomical and pathophysiological (CEAP) classification and venous clinical severity score (VCSS). We routinely assess patients with CVI on each clinical visit with CEAP and VCSS. This study was reviewed and approved as exemption from institutional ethical review committee (2020-4748-11008).

### Operation definitions

**CVI** represents a spectrum of conditions ranging from simple telangiectasia or reticular veins to more advanced stages, such as skin fibrosis and venous ulceration due to chronic venous hypertension. It is often attributed to superficial or deep valvular incompetence.

**CEAP** Classification

on CEAP classification, disease severity can be objectively categorised. The clinical component is scored from 0 to 6, with higher score indicating increasing disease severity.

C0: No visible or palpable signs of venous disease

C1: Telangiectasia or reticular veins

C2: Varicose veins

C3: Oedema

C4: Pigmentation or eczema, lipodermatosclerosis or atrophie blanche or healed venous ulcer

C5: Healed leg ulcer

C6: Active venous ulcer

### Venous clinical severity score

Disease severity can also be categorised using VCSS. It includes 10 clinical parameters, each scored on a severity scale from 0 to 3. These include pain, varicose veins, venous oedema, skin hyperpigmentation, inflammation, induration, number of active ulcers, size of ulcers and duration and use of compressive therapy for the last 3 months.

The data was collected on a specially designed pro forma which included patient’s age, gender, occupation, presenting complaints, duration of disease, any previous investigation and its finding, any previous treatment and clinical stage of CEAP and VCSS.

Patients were then divided into two groups, depending on the severity of the disease: ‘mild to moderate’ disease if they were having CEAP classification ≤C3 or VCSS <5 or ‘severe’ form of the disease if CEAP classification >3 or VCSS ≥5. Both groups were compared to determine the factors associated with ‘severe’ CVI in terms of age (≤40/41–60/>60 years), body mass index (BMI) (normal/overweight/obese), gender (male vs. female), professions (office workers/field workers/housewife), educational level (undergraduates/graduates or above) and aetiologies of CVI (primary vs. post-thrombotic).

The data was analysed using SPSS software. Quantitative variables were reported as mean±standard deviation or median depending on the distribution of the data and assessed by independent t-test/Mann–Whitney U test. Meanwhile, qualitative variables were reported as frequency and percentages and were assessed using chi-square/Fisher’s exact test as appropriate. A p-value of <0.05 was considered as significant.

## Results

During the study duration, 121 patients presented to a vascular surgery clinic with CVI with mean age of 47.83±12.02 years; 74 (61.2%) were females. Mean BMI of the patients was 32.49±18.3. In total, 65 (53.7%) patients had both legs involved. Leg pain (75, 62%) and oedema (61, 50.4%) were identified as the most common presenting symptoms. Majority of the patients (65, 53.7%) had never received any treatment for said complaints. Mean VCSS was 5.49±3.84 [range 0–16] (**Table 1**). Patients’ age, gender, BMI and educational status were found to be not associated with higher severity of the disease. However, as per our findings, it was determined that field workers were three to five times more likely to present with severe CVI compared to housewives and office workers [odds ratio (OD)=5.521 (95% confidence interval (CI); 1.37–22.260)] as per CEAP classification and [OR=2.29 (95%CI; 0.73–11.67)] VCSS (**Table 2**).

**Table 1:** Table 1 Demographic point estimation of the patients (n=121)

Variables	Point estimation
Age (years)	47.83±12.01
Body mass index (kg/m^2^)	32.49±18.3
Gender	
Male	47 (38.8%)
Female	74 (61.2%)
Marital status	
Single	8 (6.6%)
Married	113 (93.4%)
Occupation	
Housewife	71 (58.7%)
Office worker	38 (31.4%)
Field worker	12 (9.9%)
Education	
Primary/secondary	9 (7.4%)
Matric/intermediate	42 (34.7%)
Graduate and above	70 (57.9%)
Limb involved	
Unilateral	56 (46.3%)
Bilateral	65 (53.7%)
Symptoms	
Leg pain	75 (62.0%)
Varicose vein	57 (47.1%)
Edema	61 (50.4%)
Skin changes	21 (17.4%)
Ulcer	26 (21.5%)
Previous treatment taken	
None	65 (53.7%)
Compressive therapy	31 (25.6%)
Endovenous ablation	1 (0.8%)
Surgery	12 (9.9%)
Simple dressing	12 (9.9%)
VCSS score	5.49±3.84 [Range: 0–16]
Treatment advised	
Compressive therapy	70 (57.9%)
Surgery	3 (2.5%)
Endovascular ablation	27 (22.3%)
Sclerotherapy	5 (12.4%)
Daily dressing and antibiotic	15 (12.4%)
Surgery+sclerotherapy	1 (0.8%)

Results are presented as n (%) and mean±standard deviation. VCSS: Venous Clinical Severity Score

**Table 2:** Table 2 Univariate analysis factors associated with severity of disease (n=121)

Variables	Total	CEAP >3	OR [95%CI]	VCSS ≥5	OR [95%CI]
		n (%)		n (%)	
Age groups (years)					
≤40	36	14 (38.9%)	Ref	17 (47.2%)	Ref
41–60	66	28 (42.4%)	1.16 [0.51–2.65]	36 (54.5%)	1.34 [0.75–7.78]
>60	19	5 (26.3%)	0.56 [0.16–1.90]	13 (68.4%)	2.42 [0.75–7.78]
Gender					
Male	47	23 (48.9%)	1.99 [0.94–4.23]	29 (61.7%)	1.61 [0.76–3.39]
Female	74	24 (32.4%)	Ref	37 (50%)	Ref
BMI (kg/m^2^)					
18.5–24.9	14	6 (42.9%)	Ref	5 (35.7%)	Ref
25.0–29.9	35	13 (37.1%)	0.78 [0.22–2.78]	20 (57.1%)	2.4 [0.66–8.64]
30.0–34.9	29	10 (34.5%)	0.70 [0.19–2.59]	16 (55.2%)	2.21 [0.59–8.25]
≥35	43	18 (41.9%)	0.96 [0.28–3.25]	25 (58.1%)	2.50 [0.72–8.72]
Marital status					
Single	8	4 (50%)	Ref	3 (37.5%)	Ref
Married	113	43 (38.1%)	0.61 [0.15–2.85]	63 (55.8%)	2.1 [0.48–9.21]
Occupation					
Housewives	71	25 (35.2%)	Ref	36 (50.7%)	Ref
Office workers	38	13 (34.2%)	0.95 [0.42–2.19]	21 (55.3%)	1.20 [0.54–2.65]
Filed workers	12	9 (75%)	5.52 [1.37–22.26]*	9 (75%)	2.92 [0.73–11.67]
Education					
Primary/secondary	9	2 (22.2%)	Ref	3 (33.3%)	Ref
Matric/intermediate	42	21 (50%)	3.5 [0.65–18.85]	26 (61.9%)	3.25 [0.71–14.85]
Graduate and above	70	24 (34.3%)	1.82 [0.35–9.48]	37 (52.9%)	2.24 [0.52–9.68]

Chi-square and binary logistic regression were applied. CEAP: Clinical, Etiological, Anatomic and Pathophysiologic; VCSS: Venous clinical severity score; BMI: Body mass index; OR: odds ratio; CI: Confidence interval. *Significant

## Discussion

This study has focused on examining patients presenting with CVI for the first time at a tertiary care hospital in Pakistan. Most of the patients were young, females and obese; they presented with late stage CVI. Although majority of these patients were females (61.2%), males mostly presented with severe CVI. Field workers also presented with the severe form of the disease.

This study has limitations. It was from a single centre with confined number of patients. It only examined those patients who had resources and motivation to seek treatment from a tertiary care centre. There may be patients in the community suffering from CVI who either have no access to medical facilities or lack knowledge about the consequences of this disease or lack motivation to consult a doctor and be treated. Patients who are on the early stage may also be ignoring the symptoms as it may not be severely affecting their day-to-day activities. These patients may or may not be aware that disease at late stage may not only be difficult to treat but may also severely affect their quality of life.

This study gives a glimpse of the venous disease present in our population. As per our findings, it was determined that CVI mostly affects the young population with a mean age of 47.83±12.01 years, a finding consistent with other studies. In a multicentre, cross-sectional study conducted by Khan et al. examining 3000 patients presenting to primary physicians with CVI in Pakistan, the mean age was 39±13.2 years.^5)^ In a study conducted by Radhakrishnan et al. in India examining 6,350 CVI patients, the mean age was 49±13.2 years.^6)^

This study also showed that CVI was more prevalent among females, a finding consistent with Framingham study where the incidence of varicose veins was noted to be higher in women than in men.^7)^ Radhakrishnan et al. also noted that CVI was common among female patients in a study they have conducted on hospital patients. Kanchanabat et al. also supported the same findings, noting CVI to be more prevalent among females (54.4%).^8)^ However, the findings of the study conducted by Khan et al. proved otherwise, demonstrating that CVI was more prevalent in males (52.6%) compared to females (47.4%). The difference may be due to different selection criteria and different population studied (hospital vs. community).

Most of the patients in our study were obese with mean BMI of 32.49±18.3 kg/m^2^. This finding is consistent with the findings by Khan et al., whose study cohort had mean BMI of 31.51±232.74 kg/m^2^. Further, a study from Thailand found mean BMI of patients evaluated for CVI to be 26.7±5.4 kg/m^2^. Obesity was not identified to be a significant variable for the severe form of CVI.

We used two criteria to assess disease severity, both of which complemented each other. CVI was found to be severe (>C3 or VCSS>4) in more than half of the population. Our findings are consistent with other studies, particularly hospital-based ones. Radhakrishnan et al. found 40.2% of patients presented with ≥C4 disease. Further, as per the findings from a study conducted by Kanchanabat et al., it was found that 24.1% of patients presented with C4, 8.9% with C5 and 67.1% with C6. We found mean VCSS of 5.49±3.84 [range: 0–16]).

Non-venous causes of ulcer and leg swelling were excluded by clinical assessment and, where needed, by use of duplex scan. Reflux into superficial veins of greater than 0.5 s in standing position was considered as significant reflux for valvular incompetence.

CVI is a common problem which is often overlooked even by health care providers. This can be due to various clinical manifestations of this disease.^9)^ In our study, most of the patients did not received any treatment before coming to specialised clinics. More than 50% of these patients presented with advance CVI. All patients were offered definitive treatment for advance CVI, but only 25% got treatment on our facility. Most patients were paying for their treatment from their pocket and thus are free to make their own choices and decision. Some patients may have gotten treatment from other facilities, depending upon the affordability. Venotonic medications were sparingly used in these patients.

It was also noted that CVI was more common in housewives, and majority have bilateral disease. Leg pain, oedema and varicose veins were the most common presenting complaints. This finding is consistent with other studies. Field workers had severe CVI than office workers and housewives, as they were three to five times more at risk of developing severe disease. This may be related to increase ambulatory venous pressure. CVI prevalence vary among different populations; thus, utilising the recent data from other regions will be useful in characterising this disease.

There is need to raise awareness on this disease at community level; this should discuss not only the importance of early referral and consultation for this disease but also how improving one’s lifestyle (e.g., avoiding becoming obese) and work habits can minimise the risk of CVI.

## Conclusion

In conclusion, patients with CVI presenting to vascular surgery clinic in low- to middle-income countries were mostly females, young, obese and mostly at advance stage of the disease. Field workers were determined to be three to five times more likely to have severe CVI than housewives and office workers. Thus, there is need to raise awareness on this disease at community level.
